# Supplementary material for: The Effect of Psychological First Aid Training on Knowledge and Understanding about Psychosocial Support Principles: A Cluster-Randomized Controlled Trial
Source: Int J Environ Res Public Health. 2020 Jan 11;17(2):484. doi: 10.3390/ijerph17020484 (PMC7013432; doi:10.3390/ijerph17020484)
Supplement: Supplementary file 1 [file ijerph-17-00484-s001.pdf]

Table S1: Fidelity of training to original Psychological First Aid model checklist.

DOES THE COURSE INCLUDE CONTENT ON:	YES	PARTLY	NO	NOTES
1. What PFA is – e.g., “a humane, practical approach to people who recently suffered a crisis event”, listening without pressuring someone to talk, assessing needs and concerns, connecting people to other sources of support.	YES			
2. What PFA is not – e.g., not something only professionals can do, not “counselling”.	YES			
3. What factors influence how people respond to a crisis event.			NO	
4. The "who, when, and where" of PFA.	YES			
5. The four main points of helping responsibly:<br>a. Respect safety, dignity and rights of people you help;<br>b. Adapt what you do to take account of the person’s culture;<br>c. Be aware of other emergency response measures;<br>d. Look after yourself.			NO	
6. How to stay safe while offering PFA.	YES			
7. Strategies for self and team care before, during and after helping in crisis situations.			NO	

DOES THE COURSE INCLUDE CONTENT ON:	YES	PARTLY	NO	NOTES
8. Good communication skills, including:				
a. How to listen in a supportive way.				
b. Being comfortable with silence				
c. Acknowledging if someone tells you about a serious loss				
d. Respecting privacy and keeping information confidential, where appropriate to do so.				
e. Not making false promises or giving false information				
f. Not judging the person's actions or feelings.				
9. Knowing the PFA action principles of Prepare, Look, Listen and Link.				
10. How to use the 'Prepare' action principle, including:				
a. How to approach a situation safely.				
b. Learning about available services and supports in the context				
c. Learning about the crisis event				

DOES THE COURSE INCLUDE CONTENT ON:	YES	PARTLY	NO	NOTES
11. How to use the ‘Look’ action principle, including:				
a. ~				
b. Checking for people with obvious urgent basic needs				
c. How to recognise people in distress who may need support.				
d. How to recognise and assist people who are likely to need special attention.				
12. How to use the ‘Listen’ action principle, including:				
a. How to approach people who may need support.				
b. How to ask about people’s needs and concerns				
c. What to say and do in order to be helpful to someone in distress.				
d. What not to say and do when helping people affected by crisis events (so that you don’t cause further harm).				
e. Being respectful of culture, gender, age of the affected person				
f. Finding out what is most important to a person at this moment (their priorities)				
g. Acknowledging a person’s strengths				

DOES THE COURSE INCLUDE CONTENT ON:	YES	PARTLY	NO	NOTES
13. How to use the 'Link' action principle, including:				
a. Helping people access basic needs and services (including health services)				
b. Helping people cope with problems				
c. Giving information (e.g., providing factual information, being clear about what the helper does and does not know).				
d. Connecting people with loved ones and social support (in safe ways that do not risk the spread of infection)				
e. Understanding your role and the limits of your role (i.e. when to refer)				
f. How to connect and refer to other services in the context you work in.				
14. Ending one's assistance in a positive way and ensuring the person has what they need for follow-up as necessary.				
**ARE PARTICIPANTS GIVEN:**	**YES**	**PARTLY**	**NO**	**NOTES**
15. An opportunity to practice the supportive listening skills				
16. An opportunity to practice the PFA skills in a role play/ simulation				
17. Feedback on their PFA and communication skills				
18. A pre- and post-training knowledge assessment				

Table S2: Summary statistics and results from mixed model analysis of primary and secondary outcomes in Intention-To-Treat sample (n = 408).

		Descriptive statistics mean (SD)		Mixed model analysis *	
**Outcomes**	**Time point**	**PFA (n = 206)**	**Control (n = 202)**	**Difference in LS mean (95%CI) ****	***p*-value**
Knowledge retention score	Baseline	12.14 (3.47)	12.18 (3.08)		
	Post-PFA	13.64 (3.59)	12.34 (3.24)	1.26 (0.58–1.95)	0.0003
	Follow-up	13.78 (3.56)	12.19 (3.08)	1.13 (0.40–1.85)	0.0024
Scenario score	Baseline	2.88 (1.45)	2.77 (1.32)		
	Post-PFA	3.20 (1.46)	3.17 (1.37)	0.02 (−0.28–0.32)	0.88
	Follow-up	3.42 (1.52)	2.98 (1.37)	0.46 (0.16–0.76)	0.003
Professional attitude	Baseline	30.22 (5.40)	30.59 (5.28)		
	Post-PFA	31.73 (4.96)	30.53 (5.22)	0.17 (−0.85–1.19)	0.75
	Follow-up	31.07 (4.81)	30.35 (5.28)	0.72 (−0.32–1.76)	0.17
Confidence	Baseline	19.07 (4.53)	19.13 (4.44)		
	Post-PFA	19.92 (4.36)	19.40 (4.03)	0.43 (−0.44–1.31)	0.33
	Follow-up	19.43 (4.06)	19.22 (4.11)	0.17 (−0.66–1.01)	0.68
Professional quality of life	Baseline	36.49 (5.66)	36.36 (5.66)		
	Post-PFA	36.40 (5.49)	36.40 (5.53)	−0.17 (0.00–0.97)	0.83
	Follow-up	36.57 (5.89)	36.57 (5.48)	−0.04 (-1.23–1.15)	0.95

PFA = Psychological First Aid. * The mixed model included training, time, interaction between training and time as fixed effects, subject as random effect and PHU as fixed effect. ** Effect size is calculated by the difference in LS mean by standard deviation.
